# Smoking, Comorbidities, and Low Sun Exposure Are Associated with Clinical and Radiological Outcomes in Patients with Multiple Sclerosis—A Four-Year Observational Cohort Study

**DOI:** 10.3390/jcm15114270

**Published:** 2026-06-01

**Authors:** Weronika Galus, Mateusz Winder, Aleksander Jerzy Owczarek, Katarzyna Zawiślak-Fornagiel, Magdalena Kiełbowicz-Hołysz, Joanna Siuda

**Affiliations:** 1 Department of Neurology, Faculty of Medical Sciences in Katowice, Medical University of Silesia, 40-752 Katowice, Poland; kzawislak-fornagiel@sum.edu.pl (K.Z.-F.); jsiuda@sum.edu.pl (J.S.); 2 Department of Neurology with the Stroke Subunit, Prof. K. Gibiński University Clinical Centre of Medical University of Silesia in Katowice, 40-752 Katowice, Poland; magdalenakielbowicz1506@gmail.com; 3 Department of Oncological Radiology and Nuclear Medicine, Faculty of Medical Sciences in Katowice, Medical University of Silesia, 40-752 Katowice, Poland; mwinder@sum.edu.pl; 4 Department of Pathophysiology, Faculty of Medical Sciences in Katowice, Medical University of Silesia, 40-752 Katowice, Poland; aowczarek@sum.edu.pl

**Keywords:** multiple sclerosis, disease progression, real-world data, disability, EDSS, sunlight exposure, vitamin D, brain atrophy

## Abstract

**Background:** While disease-modifying therapies reduce inflammatory activity in multiple sclerosis (MS), long-term disability progression remains insufficiently controlled. Increasing evidence points to modifiable environmental and lifestyle factors—such as smoking, sun exposure, comorbidities, and obesity—as contributors to neurodegeneration and progression independent of relapse activity. **Objective:** To evaluate the associations between smoking, comorbid conditions, sun exposure, and obesity on clinical and radiological progression in patients with relapsing–remitting MS (RRMS) over a 48-month observational period. **Methods:** We performed a retrospective secondary analysis of a previously described longitudinal cohort of 132 patients with RRMS who were monitored over four years with serial assessments of EDSS, magnetic resonance imaging (MRI) inflammatory activity as gadolinium-enhancing lesions (GELs), new or enlarged T2-weighted lesions, serum 25(OH)D levels, and linear brain atrophy metrics. Sun exposure, smoking status, obesity, and comorbidity burden were recorded at each time point. **Results:** Low sun exposure was associated with higher EDSS trajectories and lower serum 25(OH)D levels (*p* < 0.01). Smoking was associated with a higher probability of GELs (*p* < 0.05), while comorbidities were associated with relapse occurrence and GELs. Obesity was associated with vitamin D insufficiency but not clearly with clinical relapse activity, GELs, or EDSS trajectories. MRI-based indices confirmed increasing brain atrophy during follow-up, particularly in patients with multiple risk factors. **Conclusions:** Our findings suggest that selected modifiable lifestyle and clinical factors are associated with distinct clinical and radiological outcomes in RRMS. Integrating sun-safe outdoor activity, smoking cessation, comorbidity management, and weight control into MS care may support comprehensive risk management alongside pharmacological therapy.

## 1. Introduction

Multiple sclerosis (MS) is a chronic, immune-mediated disorder of the central nervous system (CNS), characterized by inflammation, demyelination, axonal injury, and neurodegeneration [1]. The etiopathogenesis of MS is multifactorial, involving a complex interplay between genetic predisposition and environmental influences [2,3].

Clinically, MS most commonly manifests as relapsing–remitting multiple sclerosis (RRMS); however, progressive subtypes also occur—primary progressive multiple sclerosis (PPMS) and secondary progressive multiple sclerosis (SPMS). Emerging evidence indicates that these phenotypes lie along a single disease continuum rather than representing strictly separate subtypes [4].

The “classical” activity of MS is characterized by clinical relapses accompanied by radiological evidence of new or enlarging T2-weighted (T2-w) lesions and gadolinium-enhancing lesions (GELs) on magnetic resonance imaging (MRI). The severity of relapses and the degree of neurological impairment are commonly assessed using the Expanded Disability Status Scale (EDSS) [5]. However, it is now well established that disease progression is not solely driven by overt inflammatory activity. Longitudinal studies have described the phenomenon of silent progression, which primarily correlates with the extent of brain atrophy [6]. Increasing evidence indicates that relapse-associated worsening contributes less to long-term disability accumulation than progression independent of relapse activity (PIRA) [7].

Despite significant therapeutic progress—most notably through the advent of high-efficacy disease-modifying therapies such as anti-CD20 monoclonal antibodies, which effectively suppress classical inflammatory activity and partially attenuate PIRA—MS remains a complex and progressive disease. These treatments have transformed disease control, particularly in RRMS, but they do not fully halt the neurodegenerative component of the disease [8]. Moreover, MS is highly heterogeneous with respect to both clinical presentation and disease trajectory, and residual disease activity or disability accumulation may still occur despite ongoing treatment. Numerous patient-specific and clinical factors, including age, sex, comorbid conditions, obesity, and response to treatment, have been associated with disease activity and long-term disability [9].

In this context, potentially modifiable lifestyle and environmental factors—such as smoking, physical inactivity, obesity, low sun exposure, vitamin D deficiency and comorbidity burden—are increasingly recognized as clinically relevant modifiers of MS outcomes [10]. These factors may influence not only relapse-related inflammatory activity but also disability progression and MRI-based markers of neurodegeneration, including brain atrophy [11,12]. Their evaluation is therefore particularly relevant in treated RRMS populations, in whom disease activity may persist despite the use of DMTs.

Therefore, the aim of the present study was to evaluate associations between selected modifiable lifestyle and clinical factors—smoking, sun exposure, comorbidities, and obesity—and longitudinal clinical, biochemical, and MRI outcomes in patients with RRMS receiving DMTs. We hypothesized that smoking, low sun exposure, comorbidity burden, and obesity would be associated with greater disability accumulation, higher inflammatory disease activity, and/or more pronounced MRI-based atrophy measures despite ongoing treatment.

## 2. Materials and Methods

### 2.1. Study Design, Population and Eligibility Criteria

This study was designed as a retrospective secondary analysis of a previously described four-year real-world longitudinal cohort of patients with RRMS [13]. Accordingly, the study cohort and the underlying longitudinal clinical, biochemical, and MRI dataset overlap with the previous publication, which primarily evaluated vitamin D supplementation in relation to disease activity and brain atrophy. In contrast, the present analysis focuses on different modifiable lifestyle and clinical factors, including smoking, comorbidities, obesity/BMI, and sun exposure, and their associations with longitudinal clinical, biochemical, and MRI outcomes over a 48-month observational period. Thus, while the cohort and core outcome data have been previously described, the exposure-specific analyses presented here are new.

According to the original study protocol, patients were eligible for inclusion if they had a diagnosis of RRMS according to the 2010 or 2017 revised McDonald criteria, were aged >18 years, had an EDSS score ≤ 6.5, and had been receiving stable DMT for at least one year at baseline [13]. The original exclusion criteria included progressive MS phenotypes, including SPMS and PPMS; relapse within 4 weeks before baseline; corticosteroid use within 6 weeks before baseline; EDSS score > 6.5; pregnancy or breastfeeding; acute or chronic renal failure; history of hypercalcemia; DMT switch from platform therapy to high-efficacy treatment agents or DMT discontinuation/termination; and treatment with mitoxantrone or cyclophosphamide [13].

For the present secondary analysis, patients were required to have available baseline clinical, biochemical, MRI, and exposure data relevant to the study objectives, including data on smoking status, sun exposure, comorbidities, BMI, serum 25(OH)D concentration, EDSS, and MRI inflammatory activity. Patients who discontinued follow-up contributed available data up to their last completed visit. During follow-up, patients were censored from subsequent analyses in the event of missing serum 25(OH)D measurements, withdrawal of consent, transfer to another treatment center, pregnancy, death, DMT escalation from platform therapy to a high-efficacy treatment agent, or DMT discontinuation/termination.

Participants were followed for 48 months, with assessments performed at baseline and in consecutive time points. At every time point (baseline (T_0_), 12 months (T_1_), 24 months (T_2_), 36 months (T_3_), and 48 months (T_4_)), the following clinical and radiological outcomes were assessed: number of new relapses, neurological status expressed by EDSS, number of new/enlarged T2-w lesions, and number of GELs in MRI. The 25-hydroxyvitamin D (25(OH)D) serum levels were also measured at every time point and expressed in ng/mL.

Brain atrophy assessment was performed at baseline and 36 months of observation, according to the protocol described previously in detail [11]. The assessed parameters included frontal horn distance (FH), intercaudate distance (CC), third ventricle width (TV), inner table of the skull measured along the CC line (IT) and inner table of the skull measured at its maximum width (mIT). These measurements were used to calculate the BCR (bicaudate ratio or bicaudate index, CC/IT), Evans index (FH/mIT), and FH/CC ratio.

The analysis included demographic factors (age, sex), anthropometric data, obesity defined as BMI > 30 kg/m^2^, and lifestyle variables such as smoking and sun exposure. Smoking status was recorded as a binary variable based on patient report. Detailed information on pack-years, smoking duration, current versus former smoking, passive exposure, and changes in smoking status during follow-up was not available. Effective sunlight exposure was defined according to criteria relevant for vitamin D synthesis: spending at least 15 min outdoors daily between 10 a.m. and 3 p.m., from May to September, with uncovered forearms and lower legs, and without sunscreen use [14]. The questionnaire also included skin phototype. All participants were recruited from the same geographical region in southern Poland, which limited the variability within the cohort. Serum 25(OH)D levels were also included in the analysis. Baseline vitamin D supplementation was assessed using the patient questionnaire. The questionnaire recorded self-reported supplementation status, dose category, duration, frequency of use, seasonal versus year-round use, physician recommendation, and serum 25(OH)D monitoring. The study did not include a protocol-defined supplementation policy; vitamin D supplementation was not assigned, standardized, or modified by the study protocol. Because vitamin D supplementation was assessed only at baseline, follow-up changes in dose, discontinuation, and objective adherence were not available. Clinical factors included comorbidities (e.g., cardiovascular, metabolic, autoimmune diseases) and disease duration (years since MS diagnosis). Treatment-related variables encompassed disease-modifying therapies (DMTs), categorized as first-line or high-efficacy treatment agents (HETAs), including fingolimod, natalizumab, alemtuzumab, and ocrelizumab. Sunlight exposure, lifestyle habits, and medical history were reassessed at each follow-up visit.

### 2.2. Statistical Analysis

Statistical analysis was performed using STATISTICA 13.0 PL (Tibco Software Inc., Palo Alto, CA, USA) and R software (v 4.4.0; R Development Core Team (2008). R: A language and environment for statistical computing. R Foundation for Statistical Computing, Vienna, Austria). Statistical significance was set at a *p*-value below 0.05. All tests were two-tailed. The multivariate imputation by chained equation (MICE) was used to impute missing data. To impute univariate missing data, the predictive mean matching method was used (midastouch). Imputation was done with package ‘mice’, based on 320 imputed datasets with 5 iterations in the predictive mean matching calculation. On all imputed datasets, a proper longitudinal analysis model was used, and results were pooled. Nominal and ordinal data were expressed as percentages, while interval data were expressed as mean value ± standard deviation or 95% confidence interval (CI). Categorical variables were compared using χ^2^ or Fisher’s exact tests. Interval longitudinal data were analyzed with the mixed models for repeated measurements with contrasts as a post hoc analysis (package ‘mmrm’), while binary data were analyzed with a generalized linear model with binomial function and logit link function. Multiple comparisons were corrected with the Hochberg method.

Candidate clinical and lifestyle predictors were evaluated in longitudinal models as separate exposure-specific analyses. For each outcome, follow-up time, the candidate predictor, and the predictor-by-time interaction were included. Candidate predictors included age, sex, disease duration, BMI/obesity, treatment category, sun-exposure criterion, comorbidity status, and smoking status, as appropriate. These primary exposure-specific models were not mutually adjusted for the full set of candidate predictors and should therefore be interpreted as exploratory longitudinal association analyses.

In addition, exploratory multivariable models were fitted to assess whether baseline vitamin D supplementation was associated with study outcomes. Baseline supplementation was included as a binary predictor; the single record with unclear supplementation status was excluded from yes-versus-no models. These models were adjusted for follow-up time, age, sex, BMI > 30 kg/m^2^, sun-exposure criterion, comorbidities, smoking status, and disease duration. Continuous outcomes are reported as β coefficients and binary outcomes as odds ratios with 95% confidence intervals. Model specifications are summarized in Supplementary Table S1.

### 2.3. Ethics Approval

The study was conducted according to the guidelines of the Declaration of Helsinki and all applicable legal regulations. The study protocol was approved by the Ethics Committee of the Medical University of Silesia on 2 April 2019 (No. KNW/0022/KB/135/19), and the approval was extended on 6 February 2024 (No. BNW/NWN/0052/KB/31/24). Written informed consent was not required, according to the decision of the Ethics Committee, because the study was observational in nature, involved no intervention or experimental procedures, posed no risk to participants, and was based solely on anonymized clinical data collected during routine clinical care.

## 3. Results

Altogether, 132 patients were enrolled in the study. During follow-up, 13 patients discontinued at 24 months, a further 10 at 36 months, and another 22 at 48 months. Loss to follow-up or exclusion occurred for the following reasons: 23 patients had no vitamin D level measured, 11 patients withdrew from follow-up, five changed their treatment center, five became pregnant, and one patient died. At baseline, the mean age was 45.8 years (SD = 10.9); women accounted for 74.2% (*n* = 98) and men for 25.8% (*n* = 34). The mean disease duration was 10.0 years (range 6.0–14.0), the median Expanded Disability Status Scale (EDSS) score was 2.0 (range 1.5–6.5), and the body mass index (BMI) was 24.1 (21.9; 27.9) kg/m^2^. Comorbidities were present in 20 patients (15.2%), and 25 (18.9%) reported smoking. According to the predefined sufficient insolation criterion, 49 patients (37.1%) were classified as having sufficient sunlight exposure, whereas 83 patients (62.9%) did not meet this criterion. All patients (*n* = 132; 100%) were receiving disease-modifying therapy (DMT): interferons (*n* = 21; 15.8%), glatiramer acetate (*n* = 9; 6.8%), dimethyl fumarate (*n* = 64; 48.5%), teriflunomide (*n* = 17; 12.9%), fingolimod (*n* = 11; 8.3%), natalizumab (*n* = 8; 6.1%), ocrelizumab (*n* = 1; 0.8%), and alemtuzumab (*n* = 1; 0.8%). Platform therapies were used in 111 patients (84.1%), while HETAs were used in 21 patients (15.9%).

Baseline vitamin D supplementation was reported or imputed in 97/132 patients (73.5%), while 34/132 (25.8%) reported no supplementation and one record (0.8%) remained unclear. Among supplement users, the most common dose categories were 2000 IU/day in 36/97 patients (37.1%) and 4000 IU/day in 35/97 patients (36.1%). Daily use was reported by 68/97 patients (70.1%), supplementation for more than 6 months by 64/97 (66.0%), and year-round use by 59/97 (60.8%). Physician-recommended supplementation was reported by 72/97 patients (74.2%) and serum 25(OH)D monitoring by 71/97 patients (73.2%).

### 3.1. Follow-Up of Studied Parameters of Disease Progression

Changes in clinical, biochemical, and MRI-derived parameters in the studied group at five timepoints (T_0_–T_4_) are presented in Table 1.

Disability, assessed by the Expanded Disability Status Scale, increased gradually across visits and became statistically significant only at the final timepoint relative to baseline; earlier comparisons were not significant. The proportion of patients with clinical relapses fluctuated without a consistent pattern and did not change significantly overall, although a late upward tendency was observed.

MRI indicators of inflammatory activity rose over time: the shares of patients with new T2-weighted lesions and with gadolinium-enhancing lesions increased progressively, with significant differences emerging at the last assessment and, at most, borderline trends earlier.

Biochemically, serum 25-hydroxyvitamin D concentrations showed a modest upward trajectory with borderline evidence at later visits. Concordantly, the proportion of patients meeting a vitamin D sufficiency threshold increased over time, reaching significance before the final visit and remaining borderline thereafter.

**Figure 1:**
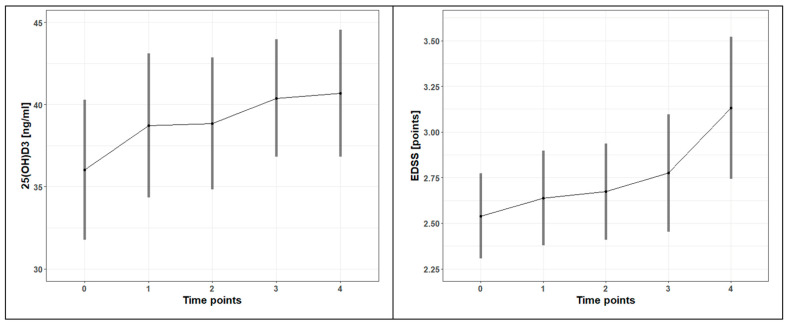
Vitamin D serum level (25(OH)D) and Expanded Disability Status Scale (EDSS) scoring through follow-up (mean values with 95% confidence intervals).

Figure 1 shows mean values with 95% confidence intervals of 25(OH)D and EDSS scale values through follow-up.

Atrophy measures showed evidence of progression from T_0_ to T_3_. CC and TV increased significantly, and CC/IT rose accordingly, indicating progressive ventricular enlargement. FH and the Evans index showed borderline increases. By contrast, IT and mIT remained stable, supporting that changes reflect parenchymal atrophy rather than cranial-size variability. FH/CC decreased significantly, consistent with proportionally greater widening at CC than at FH. Longitudinal changes in atrophy measurements (T_0_–T_3_) are presented in Table 2.

### 3.2. Correlation with Clinical Factors and Disease Progression

#### 3.2.1. Age

There were positive correlations between age and the EDSS scale (r = 0.28 (95% CI: 0.20–0.36); *p* < 0.001), CC (r = 0.21 (95% CI: 0.09–0.33); *p* < 0.01), TV (r = 0.21 (95% CI: 0.09–0.33); *p* < 0.01), and the CC to IT ratio (r = 0.24 (95% CI: 0.11–0.35); *p* < 0.001) and negative correlations with the FH to CC ratio (r = −0.31 (95% CI: −0.42–−0.18); *p* < 0.001).

#### 3.2.2. Disease Duration

There were also positive correlations between the duration of the disease and vitamin D concentrations (r = 0.16 (95% CI: 0.08–0.24); *p* < 0.001), the EDSS scale values (r = 0.22 (95% CI: 0.14–0.30); *p* < 0.001), CC (r = 0.18 (95% CI: 0.06–0.29); *p* < 0.01), and the CC to IT ratio (r = 0.19 (95% CI: 0.06–0.30); *p* < 0.01) and negative correlations with the FH to CC ratio (r = −0.31 (95% CI: −0.42–−0.19); *p* < 0.001).

#### 3.2.3. Obesity

Obese subjects were twice as likely (OR = 2.04; 95% CI: 1.25–3.33) to have improper vitamin D concentrations (51.5% (95% CI: 40.1–62.8%) vs. 34.2% (95% CI: 30.1–38.5%); *p* < 0.01).

#### 3.2.4. DMT

Also, subjects treated with HETAs were almost twice as likely (OR = 1.69; 95% CI: 1.09–2.63) to have improper vitamin D concentrations (47.0% (95% CI: 37.3–57.0%) vs. 34.4% (95% CI: 30.2–38.9%); *p* < 0.05). There was a significant influence of HETAs (*p* < 0.001) and time of follow-up (*p* < 0.01) on the EDSS scale values. In each measurement through follow-up, subjects with HETAs had higher values of the EDSS scale.

#### 3.2.5. Sunlight Exposure

**Figure 2:**
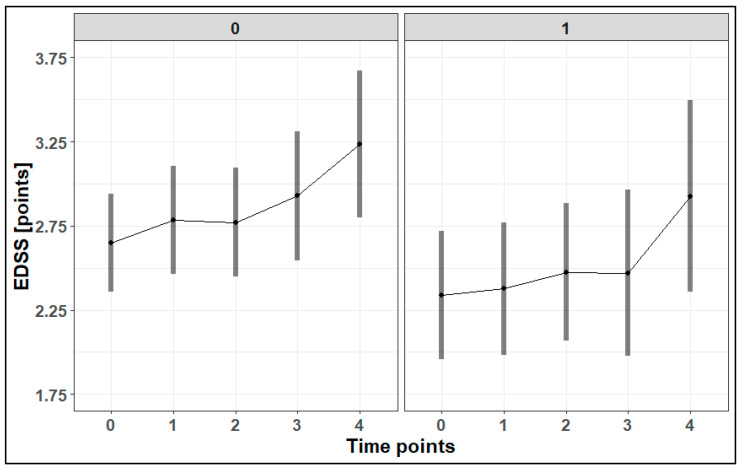
Sunlight exposure and disability progression in patients with multiple sclerosis during 4-year follow-up (EDSS—Expanded Disability Status Scale; 0—low sun exposure, 1—sufficient sun exposure).

Subjects with sun exposure were almost twice as likely (OR = 1.73; 95% CI: 1.20–2.49) to have proper vitamin D concentrations (71.4% (95% CI: 64.9–77.1%) vs. 59.0% (95% CI: 53.7–64.0%); *p* < 0.01). There was a significant influence of sun exposure (*p* < 0.001) and time of follow-up (*p* < 0.01) on the EDSS scale values. In each measurement through follow-up, subjects with sufficient sun exposure had lower values of the EDSS scale (Figure 2).

#### 3.2.6. Baseline Vitamin D Supplementation

In exploratory adjusted analyses excluding the single record with unclear supplementation status, baseline vitamin D supplementation was associated with higher serum 25(OH)D concentrations (β = 12.13 ng/mL; 95% CI: 6.40–17.86; *p* < 0.001) and a higher probability of serum 25(OH)D > 30 ng/mL (OR = 3.48; 95% CI: 1.67–7.23; *p* < 0.001). Supplementation was not significantly associated with EDSS, relapse occurrence, new T2 lesions, or morphometric atrophy indices. A nominally lower probability of gadolinium-enhancing lesions was observed among supplement users (OR = 0.51; 95% CI: 0.26–1.00; *p* = 0.048), but this finding should be interpreted cautiously because supplementation was not randomized.

#### 3.2.7. Comorbidities

**Figure 3:**
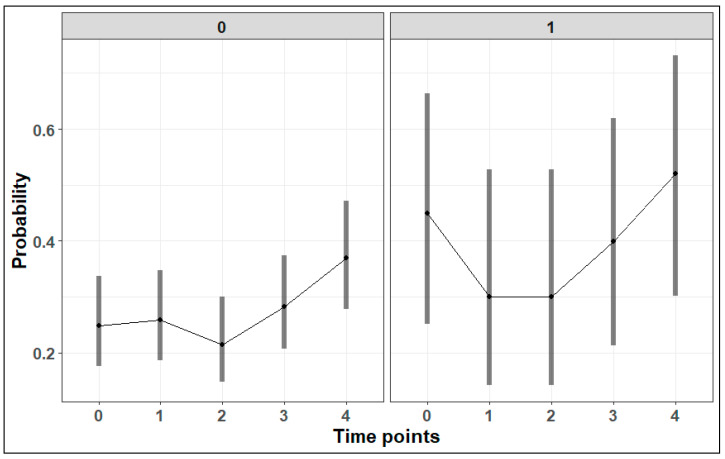
Association between comorbidity status and relapse occurrence in multiple sclerosis patients (0—without comorbidities; 1—with comorbidities).

**Figure 4:**
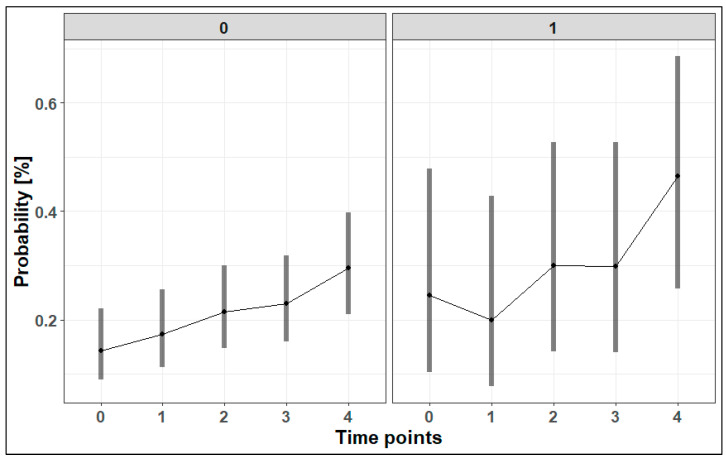
Probability of gadolinium-enhancing lesions in multiple sclerosis patients with and without comorbidities during 4-year follow-up (0—without comorbidities; 1—with comorbidities).

Subjects with comorbidities were more likely to have a relapse during the whole follow-up period (39.4% (95% CI: 30.2–49.3%) vs. 27.5% (95% CI: 23.9–31.4%); *p* < 0.05), as presented in Figure 3. Additionally, the probability of GELs was higher in patients with comorbidities through the follow-up (30.0%, 20.0%, 25.0%, 24.9%, 56.8% vs. 16.0%, 21.8%. 15.2%, 22.2%, 29.5%), as illustrated in Figure 4.

#### 3.2.8. Smoking

**Figure 5:**
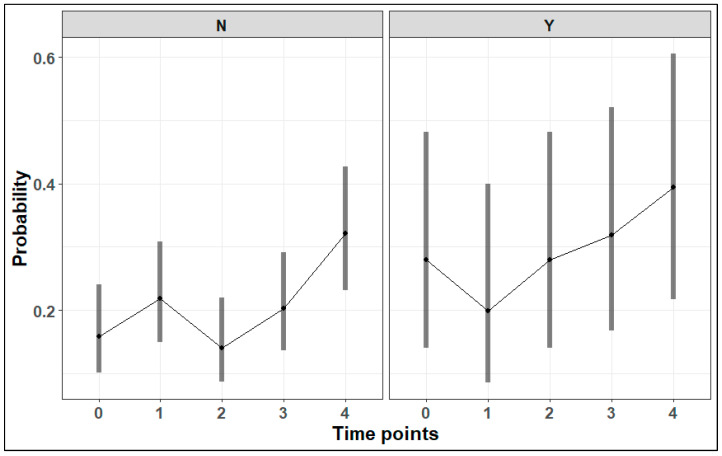
Association between smoking status and the probability of gadolinium-enhancing lesions in multiple sclerosis patients (N—non-smoking; Y—smoking).

In patients who smoked, the probability of GELs was significantly higher compared with non-smokers (29.5% [95% CI: 22.1–38.2%] vs. 20.9% [95% CI: 17.6–24.6%]; *p* < 0.05), as shown in Figure 5.

## 4. Discussion

This four-year observational study suggests that selected lifestyle and clinical factors are associated with distinct clinical and radiological outcomes in patients with RRMS. Low sun exposure was associated with higher EDSS trajectories and lower 25(OH)D levels, whereas smoking and comorbidities were mainly associated with markers of inflammatory disease activity, including GELs and/or relapses. Obesity was associated with vitamin D insufficiency but was not clearly associated with clinical relapse activity, GELs, or EDSS trajectories in the present dataset. These findings should be interpreted as associations rather than causal effects. These observations are consistent with previous evidence highlighting the role of environmental factors in MS [15].

In our cohort, patients who reported sufficient sun exposure were more likely to maintain adequate 25(OH)D concentrations and showed significantly lower EDSS trajectories over time. This supports the relevance of sun exposure as a behavioral marker related to vitamin D status and disability burden in patients with RRMS. In a Swedish registry of incident MS (*n* = 3314), very low sun exposure at diagnosis predicted faster EDSS worsening and higher hazards of confirmed disability worsening, with a graded reduction in risk across higher exposure categories [16,17,18,19,20]. However, our assessment of sun exposure was based on a simplified behavioral definition and did not capture season, weather conditions, clothing, skin phototype, occupational exposure, travel, sunscreen adherence, or actual UVB dose. Therefore, this variable should be interpreted as a crude proxy of sun exposure rather than an objective measure of ultraviolet radiation. In addition, reverse causation cannot be excluded: patients with higher disability, fatigue, heat sensitivity, or reduced mobility may spend less time outdoors, which could partly explain the observed association between lower sun exposure and higher EDSS values. Moreover, because some patients received vitamin D supplementation, sun exposure-related and vitamin D-related effects cannot be reliably disentangled. Consistently, baseline vitamin D supplementation was strongly associated with serum 25(OH)D status in exploratory adjusted analyses, but it was not consistently associated with the main clinical, MRI inflammatory, or morphometric atrophy outcomes. Therefore, supplementation should be considered an important determinant of vitamin D status and a potential confounder of sun-exposure analyses, rather than evidence of a causal effect on disease activity in this cohort.

Obesity showed a more limited pattern of association in the present cohort. Obese patients were more likely to have vitamin D insufficiency, but obesity was not clearly related to relapse occurrence, GELs, or EDSS trajectories. This finding is consistent with the view that BMI alone may not fully capture the relationship between adiposity and MS outcomes, because central adiposity, body composition, metabolic dysfunction, and longitudinal weight change may be more informative than BMI category alone [21,22]. Thus, the absence of a clear association between BMI-defined obesity and inflammatory disease activity in this cohort should not be interpreted as evidence that obesity is clinically irrelevant in MS. Rather, it indicates that within this sample and follow-up period, obesity was mainly associated with vitamin D status.

Comorbidities and smoking were more closely related to inflammatory activity than to disability trajectories in our data. These findings are consistent with previous reports suggesting that systemic health status may affect inflammatory activity, recovery potential, and disability outcomes in MS [23,24,25,26,27,28,29,30,31,32,33,34]. However, comorbidities were analyzed as a broad category, and the sample size did not allow disease-specific subgroup analyses. Therefore, these results should be interpreted as hypothesis-generating rather than as evidence for the effect of any specific comorbid condition. More detailed studies are needed to determine whether particular cardiometabolic, autoimmune, psychiatric, or other comorbidities differentially influence relapse risk, MRI activity, or disability accumulation.

Active smoking was associated with a higher probability of GELs, suggesting a relationship with focal MRI inflammatory activity. This observation is consistent with previous evidence linking smoking to increased inflammatory lesion burden and worse MS outcomes [31,32,33,34,35]. Nevertheless, smoking was recorded as a binary variable, and detailed information on pack-years, smoking duration, current versus former smoking, passive exposure, and changes in smoking status during follow-up was not available. Therefore, the strength and dose–response nature of this association could not be assessed. Despite these limitations, the observed association supports the importance of smoking cessation counseling as part of routine MS care.

The clinical relevance of these findings lies in their potential contribution to comprehensive MS care. The results support routine attention to sun-safe outdoor activity, smoking cessation, and comorbidity management alongside optimized DMT. However, these recommendations should be framed as general health and risk-management strategies rather than as interventions proven by this study to modify disease course. Our findings also suggest that different exposures may relate to different dimensions of MS-related outcomes: low sun exposure was mainly associated with EDSS trajectories and vitamin D status, smoking with MRI inflammatory activity, comorbidities with relapses and GELs, and obesity with vitamin D insufficiency. This distinction is important because these factors should not be interpreted as uniform predictors of progression.

Comprehensive MS care should also consider cognition, quality of life, and psychological well-being. Cognitive impairment is common in MS, and recent evidence suggests that cognitive re-education and rehabilitation may improve selected cognitive domains, although effects on quality of life and psychological well-being are variable [36]. These outcomes were not assessed in our cohort but remain relevant to multidisciplinary MS management.

### Study Strengths and Limitations

This study has several strengths. First, it provides four-year real-world longitudinal data on RRMS patients receiving DMTs. Second, repeated clinical, biochemical, and MRI assessment allowed the evaluation of several disease-related outcomes, including EDSS trajectories, relapses, GELs, new/enlarged T2-w lesions, serum 25(OH)D status, and MRI-based linear atrophy measures. Third, the study assessed several modifiable lifestyle and clinical factors within one analytical framework, including sun exposure, smoking, obesity and comorbidity burden. Finally, the combination of clinical and MRI outcomes enables assessment of both inflammatory activity and structural changes over time. Taken together, the main added value of this analysis is its real-world longitudinal assessment of potentially modifiable factors as multidimensional correlates of RRMS outcomes, integrating clinical disability, inflammatory MRI activity, vitamin D status, and MRI-based atrophy measures within a single analytical framework.

Several limitations should also be acknowledged. The retrospective secondary-analysis design and single-center setting limit causal inference and may introduce selection bias. The moderate sample size and missing data may have affected the robustness of subgroup analyses. Lifestyle variables were self-reported or simplified, particularly sun exposure and smoking status. Sun exposure was assessed using a simple self-reported criterion related to vitamin D synthesis; therefore, it should be considered only an approximate measure of sun exposure, not an objective measure of UVB dose, and some misclassification may have occurred. Reverse causation cannot be excluded, as lower sun exposure may reflect higher disability and related limitations rather than independently contribute to EDSS worsening. Comorbidities were analyzed as a broad category, which prevented the evaluation of specific conditions. Obesity was defined using BMI, without measures of central adiposity, body composition or metabolic markers. Brain atrophy was assessed using linear MRI indices rather than volumetric three-dimensional methods, limiting the precision of neurodegeneration estimates. Additionally, vitamin D supplementation was another relevant potential confounder. Vitamin D supplementation was self-reported and assessed only at baseline. No standardized supplementation policy or follow-up data on dose changes or adherence were available; therefore, residual confounding cannot be excluded. Finally, residual confounding remains possible, including by baseline disability, disease severity, treatment category, physical activity, and other health-related behaviors.

## 5. Conclusions

In this four-year observational cohort of patients with RRMS treated with DMTs, selected lifestyle and clinical factors were associated with distinct clinical, biochemical, and radiological outcomes. Low sun exposure was associated with higher EDSS trajectories and lower serum 25(OH)D levels. Smoking was associated with a higher probability of gadolinium-enhancing lesions, whereas comorbidities were associated with relapse occurrence and GELs. Obesity was associated with vitamin D insufficiency but was not clearly associated with clinical or MRI inflammatory activity in this cohort.

These findings support the relevance of modifiable lifestyle and clinical factors in comprehensive MS care. However, because of the observational design, single-center setting, attrition, self-reported exposure assessment, possible vitamin D supplementation effects, residual confounding, and reverse causation, the results should be interpreted as associations rather than evidence of causal effects.

## Figures and Tables

**Table 1:** Longitudinal changes in clinical, biochemical, and MRI-derived parameters in patients with relapsing–remitting multiple sclerosis at five timepoints, T_0_–T_4_.

	T_0_	T_1_	T_2_	T_3_	T_4_	*p* (T_1_–T_0_)	*p* (T_2_–T_0_)	*p* (T_3_–T_0_)	*p* (T_4_–T_0_)	Δ_4–0_
EDSS [pts]	2.54 (2.30–2.77)	2.63 (2.38–2.89)	2.67 (2.41–2.93)	2.77 (2.44–3.08)	3.12 (2.75–3.48)	0.215	0.104	0.070	<0.001	0.58 ^#^ (0.27–0.90)
Relapse (Yes vs. No) [%]	27.9 (20.8–36.2)	26.5 (19.7–34.7)	22.7 (16.4–30.6)	30.0 (22.8–38.4)	39.3 (30.7–48.7)	0.806	0.340	0.704	0.061	–
New T2-w [%]	15.8 (10.5–23.1)	17.7 (12.1–25.3)	22.7 (16.4–30.6)	24.1 (17.5–32.2)	32.1 (23.9–41.5)	0.676	0.159	0.098	<0.01	–
GELs [%]	18.1 (12.4–25.5)	21.5 (15.3–29.4)	16.7 (11.2–24.0)	22.5 (16.2–30.5)	33.7 (25.4–43.1)	0.490	0.753	0.374	<0.01	–
25(OH)D [ng/mL]	36.0 (31.8–40.3)	38.7 (34.4–43.1)	38.9 (34.9–43.0)	40.4 (36.8–44.0)	40.6 (36.8–44.5)	0.159	0.184	0.061	0.058	4.60 ^$^ (−0.18–9.39)
25(OH)D > 30 [ng/mL]	55.3 (46.7–63.6)	60.2 (51.3–68.6)	65.9 (57.0–73.9)	68.3 (59.0–76.4)	68.1 (57.6–77.0)	0.428	0.087	<0.05	0.060	–

EDSS—Expanded Disability Status Scale; T2-w—T2-weighted; GELs—gadolinium-enhancing lesions; 25(OH)D—25-hydroxyvitamin D; ^$^ *p* < 0.1; ^#^ *p* < 0.001.

**Table 2:** Longitudinal changes in atrophy parameters in patients with relapsing–remitting multiple sclerosis between T_0_ and T_3_.

	T_0_	T_3_	*p* (T_3_–T_0_)	Δ_4–0_
FH	34.3 (33.5–35.1)	34.7 (33.8–35.6)	0.068	0.45 ^$^ (−0.03–0.94)
CC	11.3 (10.8–11.8)	11.8 (11.2–12.3)	<0.05	0.42 * (0.08–0.76)
IT	118.0 (117.0–119.0)	118.0 (117.0–119.0)	0.750	0.12 (−0.63–0.87)
mIT	133.0 (132.0–134.0)	134.0 (133.0–135.0)	0.332	0.34 (−0.35–1.02)
TV	7.38 (6.94–7.82)	7.72 (7.24–8.21)	<0.05	0.35 * (0.07–0.63)
Evans	0.257 (0.252–0.262)	0.260 (0.254–0.266)	0.071	0.003 ^$^ (−0.001–0.006)
CC/IT	0.096 (0.092–0.100)	0.100 (0.095–0.104)	<0.01	0.003 ** (0.001–0.006)
FH/CC	3.14 (3.04–3.23)	3.06 (2.96–3.16)	<0.05	−0.072 * (−0.144–−0.001)

FH—frontal horn distance; CC—intercaudate distance; IT—inner table of the skull measured along the CC line; mIT—inner table of the skull measured at its maximum width; TV—third ventricle width; Evans—Evans index; ^$^ *p* < 0.1; * *p* < 0.05; ** *p* < 0.01.

## Data Availability

The data presented in this study are not publicly available due to privacy restrictions.

## Supplementary Materials

The following supporting information can be downloaded at: https://www.mdpi.com/article/10.3390/jcm15114270/s1, Table S1: Model specifications used in the longitudinal analyses.
