# Supplementary material for: Smoking, Comorbidities, and Low Sun Exposure Are Associated with Clinical and Radiological Outcomes in Patients with Multiple Sclerosis—A Four-Year Observational Cohort Study
Source: J Clin Med. 2026 Jun 1;15(11):4270. doi: 10.3390/jcm15114270 (PMC13257719; doi:10.3390/jcm15114270)
Supplement: Supplementary file 1 [file jcm-15-04270-s001.zip › jcm-4325764-supplementary.pdf]

Supplementary Table S1. Model specifications used in the longitudinal analyses.

Analysis	Outcomes	Model family	Model terms / adjustment
Overall longitudinal change	EDSS, serum 25(OH)D, 25(OH)D >30 ng/mL, relapse occurrence, new/enlarged T2-w lesions, GELs, and MRI atrophy indices	Mixed models for repeated measurements for continuous outcomes; generalized linear models with binomial distribution and logit link for binary outcomes	Follow-up time; pairwise contrasts versus baseline where applicable
Primary candidate predictor analyses	EDSS, serum 25(OH)D, 25(OH)D >30 ng/mL, relapse occurrence, new/enlarged T2-w lesions, GELs, and MRI atrophy indices	Mixed models for repeated measurements or binomial generalized linear models, depending on outcome type	Separate model for each candidate predictor: follow-up time + candidate predictor + follow-up time × candidate predictor. These models were not mutually adjusted for the full candidate predictor set
Candidate predictors tested	—	—	Age, sex, disease duration, BMI/obesity, treatment category, sun-exposure criterion, comorbidity status, and smoking status, as applicable
Exploratory baseline vitamin D supplementation analyses	Serum 25(OH)D, 25(OH)D >30 ng/mL, EDSS, relapse occurrence, new/enlarged T2-w lesions, GELs, and MRI atrophy indices	Mixed models for repeated measurements or binomial generalized linear models, depending on outcome type	Baseline supplementation + follow-up time + age + sex + BMI >30 kg/m² + sun-exposure criterion + comorbidities + smoking status + disease duration. The single record with unclear supplementation status was excluded
